# Kinematics of the Reach-to-Grasp Movement in Vascular Parkinsonism: A Comparison with Idiopathic Parkinson’s Disease Patients

**DOI:** 10.3389/fneur.2014.00075

**Published:** 2014-05-16

**Authors:** Valentina Parma, Debora Zanatto, Elisa Straulino, Tomaso Scaravilli, Umberto Castiello

**Affiliations:** ^1^Department of General Psychology, University of Padova, Padova, Italy; ^2^Unità Operativa di Neurologia, Ospedale dell’Angelo, USL12, Mestre, Italy; ^3^Centro di Neuroscienze Cognitive, University of Padova, Padova, Italy

**Keywords:** bradykinesia, hypometria, idiopathic Parkinson’s disease, kinematics, reach-to-grasp, vascular parkinsonism

## Abstract

The performance of patients with vascular parkinsonism (VPD) on a reach-to-grasp task was compared with that of patients affected by idiopathic Parkinson’s disease (IPD) and age-matched control subjects. The aim of the study was to determine how patients with VPD and IPD compare at the level of the kinematic organization of prehensile actions. We examined how subjects concurrently executed the transport and grasp components of reach-to-grasp movements when grasping differently sized objects. When comparing both VPD and IPD groups to control subjects, all patients showed longer movement duration and smaller hand opening, reflecting bradykinesia and hypometria, respectively. Furthermore, for all patients, the onset of the manipulation component was delayed with respect to the onset of the transport component. However, for patients with VPD this delay was significantly smaller than that found for the IPD group. It is proposed that this reflects a deficit – which is moderate for VPD as compared to IPD patients – in the simultaneous (or sequential) implementation of different segments of a complex movement. Altogether these findings suggest that kinematic analysis of reach-to-grasp movement has the ability to provide potential instruments to characterize different forms of parkinsonism.

## Introduction

In both behavioral and neural terms, human reach-to-grasp behavior can be dissociated into separate transport and grip components (1–6). In the first instance, kinematic analysis of the reaching phase shows that during the transport of the hand toward the object, the fingers begin to pre-shape, by progressively opening the grip with straighten fingers and subsequently by closing the grip until it matches the object size. The analysis of the grasping phase confirms that key landmarks, such as the point in time in which grip size is the largest (maximum grip aperture) occurs well before the fingers come into contact with the object, indicating that the motor configuration that is formed by the hand in contact with the object represents the end result of a motor sequence that begins well ahead of the action of grasping itself (7–11). In the second instance, neural computations regarding the reach component occur within the medial intraparietal and the superior parieto-occipital cortex (2, 5) whereas the neural underpinnings of the grasp component occur within a lateral parieto-frontal circuit involving the anterior intraparietal area and both the dorsal and the ventral premotor areas (12).

While there is an extensive literature demonstrating the key roles of fronto-parietal networks in reaching for and grasping objects (6, 13–15), there are less studies examining the role played by subcortical structures – such as the basal ganglia – during the performance of similar tasks in humans (16). An important perspective on the role of cortico-basal ganglia circuits in the unfolding of the reach-to-grasp movement have so far come from the study of patients with idiopathic Parkinson’s disease (IPD), wherein reduced tonic levels of dopamine in midbrain neurons results in a disrupted functionality of the thalamocortical–basal ganglia circuit, which is responsible for the motor irregularities (17, 18). It has been suggested that upper-limb motor deficits in IPD can be decomposed into at least two major aspects, namely intensive (amplitude, speed) and coordinative [integration and/or coordination of multiple movement components; (19–23)]. As for the intensive performance, the evidence indicates an absolute slower implementation of actions with respect to healthy controls (HC), but no shortfalls in the ability to modify the spatiotemporal characteristics of the prehension pattern in response to experimentally imposed changes (19). Individuals diagnosed with IPD are thus able to correctly regulate movement parameters and the overall form of the motor program appears to be maintained (24). Rather, it was the coordinated activation of the two components that revealed abnormalities in patients diagnosed with IPD. For instance, the onset of the grasping component was delayed with respect to the onset of the reaching component (19, 20). These results suggest that the grasping deficit shown by patients diagnosed with IPD in the activation of concurrent motor programs apply not only to the motor programs that are completely independent, but also to those only largely independent, which do show functional coordination.

The evidence so far reviewed refers to studies comparing the performance of patients diagnosed with IPD with neurologically healthy participants. To date, still little is known on how other forms of Parkinsonism impact on the kinematic organization of reach-to-grasp movements, especially the forms linked to cortico-basal degeneration. Among these syndromes there is vascular parkinsonism [VPD; (25)], a clinically heterogeneous syndrome that can be separated from IPD on the basis of the presence of additional focal signs, the absence of three typical signs, namely resting tremor in the upper limbs, true akinesia, and definite benefit from levodopa assumption (26). The lesions responsible for VPD are mostly basal ganglia lacunes and/or subcortical white matter vasculopathy of the Binswanger’s type (27, 28). In rare cases, a single striatal infarct, striatal cribriform cavities, or ischemic changes in the substantia nigra have induced this type of parkinsonism (29). All in all, the pathophysiology of VPD is still poorly understood and we are not able to fully explain the reason why, despite same apparent lesion loads, some patients do develop parkinsonism while others do not. Therefore, it appears crucial to explore alternative markers with the goal of facilitating the characterization of this disorder.

With respect to motor assessment, gait disorders have primarily been considered and characterized in the VPD population, mostly because reminiscent of – nevertheless distinct from – the gait issues found in patients with IPD (30, 31). Typically, the gait is wide-based, marked by start and turn hesitation as well as by slow and short shuffling steps (32). To refer to such motor problems, terms like “lower body,” “lower half” parkinsonism, or “frontal-type” gait disorders have been forged (30). Conversely, in terms of upper-limb movements, minimal or no dysfunctions have been reported. To date, available literature is suggestive of no true upper-limb akinesia or resting tremor, and preserved arm swing (26, 33). A point worth noting, however, is that such conclusions have been drawn on the basis of observational studies and no thorough kinematical investigations of upper-limb movements in VPD patients has been conducted.

Indeed, a close inspection of the causes underlying gait deficits in VPD might provide the ground for investigating more exhaustively upper-limb movements in this population. Gait problems in VPD are largely caused by ischemic damage to the “motor cortex–basal ganglia” and “frontal cortex–basal ganglia” connections (33). An aspect limiting the ability of central motor control systems to generate appropriately modulated descending commands. Because the above-mentioned connections are also relevant for the coordination of upper-limb movements, pathological descending signals might also affect the unfolding of this kind of actions.

In the attempt to further delineating upper-limb movements and to explore this coordinative aspect of motor control in patients with VPD, in the present study we asked a group of patients with VPD to carry out reach-to-grasp movements in the direction of visual targets of different sizes. The performance of these patients was then compared with that of a matched group of patients with IPD and with a group of neurologically HC.

Because no previous reach-to-grasp kinematical analysis on patients with VPD has been performed, only tentative predictions are advanced. First, on the basis of previous reports of pyramidal slowing (that might qualify for the term bradykinesia), a slowness of movement might be foreseen (34). Second, assuming that VPD performance is in line with that of patients with IPD, a modification of the amplification of hand opening in relation to the size of the object might be expected. Third, given the difficulties expressed by patients with VPD in coordinating gait, a dysfunction in activating almost simultaneously motor plans might be evident and emerge also at the level of the coordination of the transport and prehension components of the reach-to-grasp movements. Other aspects of reach-to-grasp kinematic parameterization are estimated to be largely unaltered with respect to neurologically healthy participants (19, 24).

## Materials and Methods

### Participants

Three groups of participants were recruited for the study. The first group (*N* = 12) was composed of patients with VPD. Demographic information, clinical data, vascular risk factors (35), and imaging details for these patients are outlined in Table 1. Participants in the second group (*N* = 12) were all diagnosed with IPD and were treated with dopaminergic drugs (Table 2). Patients with vascular lesions detected on magnetic resonance imaging (MRI) were excluded from the study with the exception of those with minimal evidence of small vessel disease considered normal for the patient’s age and in areas other than the basal ganglia (36). An independent radiologist, blinded to the study design and modality, evaluated the scans. The severity of Parkinson’s disease symptoms in both groups of patients studied was assessed by a board-certified neurologist using two different measures: the Hoehn and Yahr (37) severity scale and the Unified Parkinson’s Disease Rating Scale (38). All of the patients with IPD and three patients with VPD were tested after they had taken their medication. The fact that levodopa was producing optimal therapeutic responses was provided by the UPDRS, which was administered to those patients prior to their respective experimental session. None of the participants showed therapy-related motor complications that could interfere with the study task. A third group (*N* = 12) was made up of healthy participants (HC) without neurological or skeletomotor dysfunctions. The Mini-Mental State Examination (MMSE) was used to provide an index of the patients’ current global cognitive state (39). The scores of the patients with VPD and IPD ranged between 28 and 30 (Tables 1 and 2) while all the HC participants had a score of 30, all falling within a normal range of cognitive functioning. Mean age was not significantly different in the groups studied nor significant differences in terms of disease duration in the two groups of patients were highlighted. Both the IPD and VPD patients scored an average of 18 out of 20 on visual acuity test, while the participants in the HC scored 20 out of 20. All the participants showed right-handed dominance (40). The experimental session was individual and lasted an hour. Approved by the ethics committee of the University of Padova, this study was carried out in accordance with the principles of the Declaration of Helsinki. Written informed consent was obtained from all of the participants.

**Table 1:** **Demographic data and clinical features of the patients with vascular parkinsonism (VPD) studied**.

PD patient	Age (years)	Sex	Years since diagnosis	Most affected upper-limb	UPDRS (upper-limb)	UPSRR score	MMSE score	Clinical signs						
								T	R	B	A	P	O	F
1	66	F	3	L	4.4	35	30	−	−	−	−	−	−	−
2	68	F	3	L	3.3	37	30	−	−	−	−	−	−	−
3	68	F	2	L	6	31	30	−	−	+	−	−	−	−
4	69	F	4	L	4.8	34	30	R	−	+	−	−	−	−
5	69	F	1	L	3	33	29	−	−	+	−	−	−	−
6	70	F	3	R	8	36	29	L	−	+	−	−	−	−
7	72	F	2	L	3	35	28	R	+	+	−	−	−	−
8	68	M	2	L	6.2	32	29	L	−	−	−	−	−	−
9	66	M	4	R	5	36	30	L	+	+	+	−	−	−
10	67	M	2	L	10	34	29	R	−	+	+	−	−	−
11	69	M	3	L	4	37	30	−	−	L	−	−	−	−
12	71	M	2	L	8	35	30	−	−	+	+	−	−	−

Patient	Onset	Clinical features	MRI	Vascular risk factors	l-DOPA response
1	Insidious	Hemiparkinsonism following stroke, bradykinesia	DWML, PWML	Hypertension	Not tried
2	Insidious	Asymmetric parkinsonism with tremor, bradykinesia	DWML, PWML	Hypertension	Good
3	Acute	Hemiparkinsonism following stroke, bradykinesia	Lesion contralateral LN	Hypertension	Not tried
4	Acute	Asymmetric parkinsonism with tremor, bradykinesia	Bilateral GP lesion	Hypertension, stroke	Not tried
5	Acute	Hemiparkinsonism following stroke, bradykinesia	Lesion contralateral GP	Stroke	Not tried
6	Acute	Hemiparkinsonism following stroke, bradykinesia	Bilateral GP lesion	Hypertension, stroke	Poor
7	Insidious	Hemiparkinsonism following stroke, bradykinesia	DWML, PWML	Family history of stroke	Good
8	Acute	Hemiparkinsonism following stroke, bradykinesia	Bilateral GP lesion	Hypertension, diabetes	Not tried
9	Acute	Shuffling gate, bradykinesia	Lesion contralateral LN	Stroke	Not tried
10	Insidious	Lower body parkinsonism, bradykinesia	DWML, PWML	Family history of stroke	Good
11	Insidious	Shuffling gate, asymmetrical Parkinsonism with rest tremor, bradykinesia	DWML, PWML	Hypertension	Good
12	Acute	Hemiparkinsonism following stroke, bradykinesia	Lesion contralateral GP	Stroke	Not tried

**Table 2:** **Demographic data and clinical features of the patients with idiopathic Parkinson’s disease (IPD) studied**.

PD patient	Age (years)	Sex	Years since diagnosis	Stage of the disease	Most affected upper-limb	UPDRS (upper-limb)	UPSIT score	MMSE score	Dopaminergic medication	Clinical signs						
										T	R	B	A	P	O	F
1	65	F	4	II	L	4	18	30	0–0–0	−	+	+	−	−	−	−
2	66	F	1	II	L	9	15	30	0.5–0.5–0.5^b^	−	−	+	+	−	−	−
3	68	F	2	II	R	8	14	30	1–1–1^a^	−	−	+	+	−	−	−
4	68	F	3	I	R	5	15	29	0–0–0	−	−	R	L	−	−	−
5	71	F	1	I	R	6	14	30	0–0–0	−	+	R	−	−	−	−
6	71	F	2	II	L	12	13	30	1–1–1	R	R	+	+	−	−	−
7	66	M	3	II	L	2	17	28	0–0–0	−	−	+	+	−	−	−
8	66	M	3	II	L	10	17	29	1–1–1^a^	−	+	R	+	−	−	−
9	67	M	2	II	L	5	17	30	1–0–1^a^	−	+	+	+	−	−	−
10	68	M	2	I	L	3	15	30	1–1–1^a^	R	+	+	+	−	−	−
11	68	M	3	I	L	2	17	30	0–0–0	−	−	R	−	−	−	−
12	69	M	2	I	R	8	12	30	0–0–0	−	−	+	−	−	−	−

*Medication: number of tablets, morning–midday–evening (dopaminergic medication, ^a^50 mg; ^b^125 mg). Clinical signs: signs when medicated, according to examination at time of testing and self report: T, resting and/or postural tremor; R, rigidity; B, bradykinesia; A, akinesia; P, problems with static and dynamic upright posture; O, on–off phenomenon; F, freezing; “+,” both sides affected; “−,” neither side noticeably affected; L, left side mainly affected; R, right side mainly affected; MMSE, Mini-Mental State Examination. Stage of the disease was determined on the basis of the Hoehn and Yahr’s scale*.

### Stimuli and apparatus

**Figure 1:**
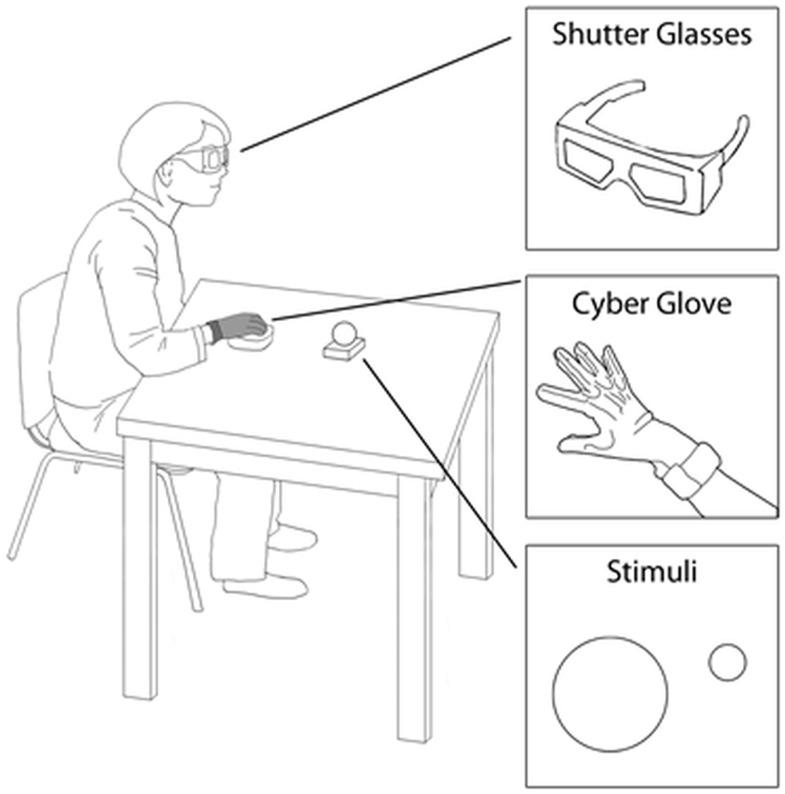
**Graphical representation of the experimental set-up**. Legends indicate the relevant details.

The visual stimuli (i.e., to-be-grasped targets) consisted of two plastic spherical objects (small object = 4 cm diameter; large object = 8 cm diameter). At the beginning of the session, each individual was asked to place his/her right hand on a starting platform within which a pressure sensitive switch was embedded (i.e., starting switch). The platform was designed with slight convexities dictating a natural flexed posture of the fingers (Figure 1). The target object was placed on a second pressure sensitive switch (i.e., the ending switch) embedded within the working surface (Figure 1). To control vision, the participants were asked to wear spectacles fitted with liquid crystal lenses (Translucent Technologies Inc., Toronto, ON, Canada), able to change from opaque to transparent (Figure 1). Participants were told that pressing the starting switch, which would determine visual availability of the target (i.e., opening of the spectacles), should correspond to the onset of the reaching movement toward the target.

### Recording techniques

Hand kinematics was measured by means of a flex sensor glove (CyberGlove, Virtual Technologies, Palo Alto, CA, USA), worn on the participant’s right hand (Figure 1). The sensors’ linearity was 0.62% of maximum non-linearity over the full range of hand motion. The sensors’ resolution was 0.5° remaining constant over the entire range of joint motion. The output of the transducers was sampled at 12-ms intervals.

### Procedures

At the beginning of the session, the participant was positioned with his/her elbow and wrist resting on a flat surface, the forearm horizontal, the arm was oriented in a natural parasagittal plane passing through the shoulder, and the right hand was placed in a pronated position with the palm toward the working surface on the starting switch. The target was aligned with the participant’s body midline, located 33 cm from the hand starting position to the left of the participant’s right shoulder (Figure 1). The sequence of events for each trial was the following: (1) once correctly positioned, the participant’s vision was occluded while the target was being placed on the working surface; (2) 500 ms later an auditory signal was sounded; (3) participants were instructed to reach toward, grasp, and lift the target when they heard the tone. The participants were instructed to reach for the object at a natural speed. An experimenter visually monitored all the trials to ensure that participants complied with instructions. The experimenter noted that the participants naturally grasped the small objects between the thumb and the index finger, at times also with the help of the middle fingers, while the large objects were grasped using the thumb and the rest of the fingers. The task was performed under two experimental conditions: (i) a reach-to-grasp movement toward the large target (“large” condition); and (ii) a reach-to-grasp movement toward the small target. Each participant took part in a total of 48 trials (24 for each experimental condition), which were presented in randomized order.

### Dependent measures

In accordance with previous reports assessing the kinematics of reach-to-grasp movements in patients with IPD, the dependent variables specifically relevant to test our hypotheses were: (i) *movement time*, namely the time occurring from the release of the starting switch and the time at which the hand closed upon the object, to test for the slowness in movements in patients with Parkinson’s disease; (ii) *maximum grip aperture*, or the amplitude of the maximum distance reached by the index finger and thumb in the transport phase, to test for hand opening alterations [hypometria; (41)]; and (iii) *delay*, or the interval between the beginning of the arm movement and the opening of the fingers, to test for impaired coordination of the reach and grasp components (19).

### Data analysis

For each dependent measure, a mixed analysis of variance (ANOVA) with “target size” (small, large) as within-subjects factor and “group” as between-subjects factor (VPD, IPD, HC) was performed. The main assumptions behind this statistical model (i.e., normality and sphericity) were checked before running the ANOVA. The Kolmogorov–Smirnov test showed that the normality assumption was satisfied (α-level: *p* < 0.05). The Mauchly test showed that the sphericity assumption was not violated. Results from the ANOVA performed on the slope absolute values were assessed through *post hoc* comparisons using *t*-tests. The Bonferroni’s correction was applied whenever required (α-level: *p* < 0.05).

## Results

### Movement time

**Figure 2:**
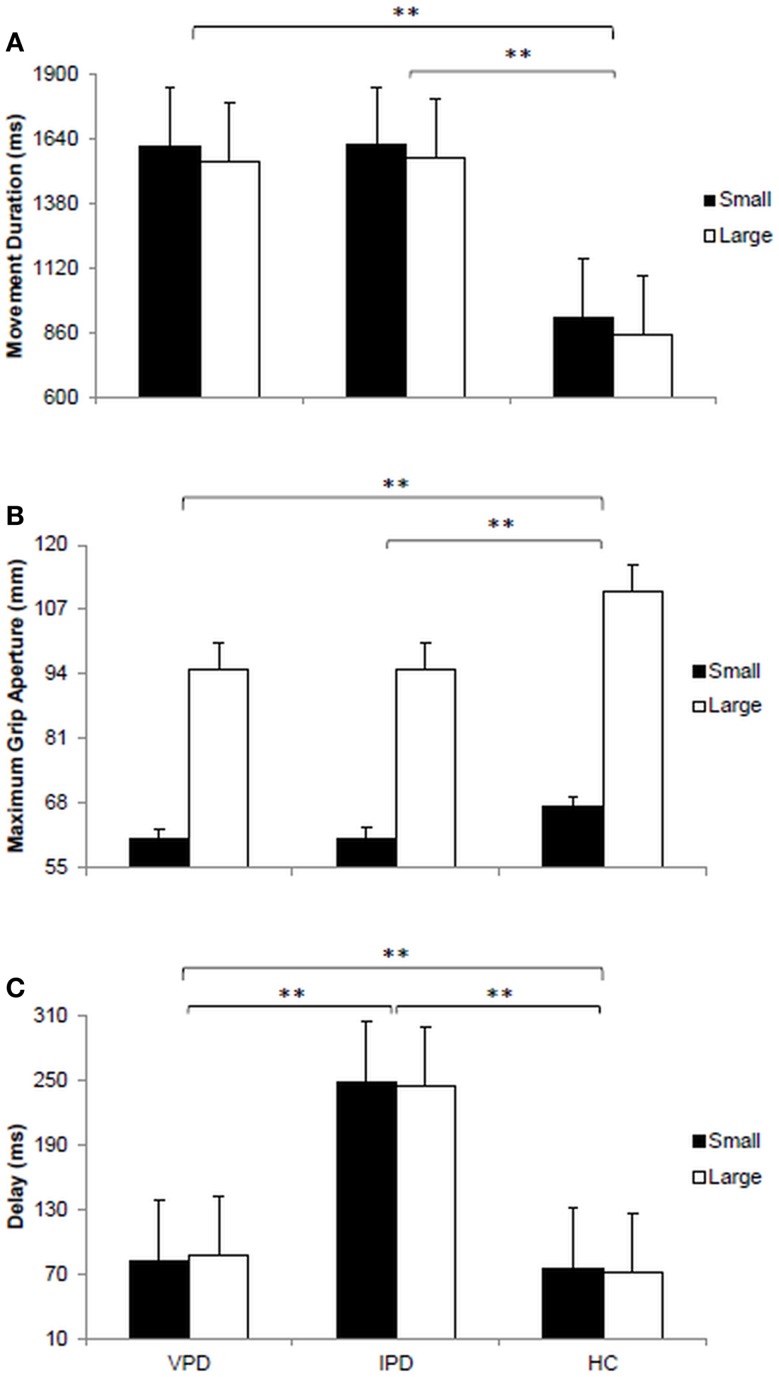
**Visual presentation of the dependent variables measured for each of the groups tested**. **(A)** Bar plot represents the movement duration expressed in milliseconds (ms). **(B)** Bar plot shows the maximum grip aperture measured in millimeters (mm). **(C)** Bar plot demonstrates the delay between the beginning of the arm movement towards the target object and the opening of the fingers to grasp it. VPD, vascular parkinsonism; IPD, idiopathic Parkinson’s disease; HC, healthy controls.

The main effect of “target size” was significant for movement duration [*F*(1, 11) = 388.92, *p* < 0.0001, η*p*^2^ = 0.972]. For all groups movements toward the small stimulus were longer than those toward the large stimulus (1385 ± 180 vs. 1322 ± 109 ms). The main effect of “group” was significant for movement duration [*F*(2, 11) = 159.76, *p* < 0.0001, η*p*^2^ = 0.936; Figure 2A]. *Post hoc* contrasts indicate that movement duration for the VPD group (1581 ± 45 ms) was comparable to that of the IPD group (1593 ± 38 ms), and both longer than for the HC group (887 ± 52 ms; *p*s < 0.05). No significant two-way interaction “target size” by “group” was found (*p*s > 0.05).

### Maximum grip aperture

The main effect of “target size” was significant [*F*(1, 11) = 919.96, *p* < 0.0001, η*p*^2^ = 0.988]. Participants’ maximum grip aperture was larger for the large target as compared to the small target (100 ± 9 vs. 63 ± 4 mm). Significant differences across groups were also evident [*F*(2, 11) = 78.11, *p* < 0.0001, η*p*^2^ = 0.877; Figure 2B]. *Post hoc* contrasts revealed that the amplitude of maximum grip aperture was significantly larger for the HC participants (87 ± 31 mm) than for both the VPD (78 ± 24 mm) and the IPD groups (78 ± 24 mm; *p*s > 0.05). A significant two-way interaction “target size” by “group” was found [*F*(2, 11) = 72.99, *p* < 0.0001, η_p_^2^ = 0. 869]. For the large target, maximum grip aperture was larger for HC participants (111 ± 2 mm) than for both the VPD (95 ± 5 mm) and the IPD groups (95 ± 5 mm; *p*s > 0.05). Moreover, for the small target, maximum grip aperture was larger for HC participants (67 ± 3 mm) than for the IPD group (61 ± 6 mm; *p* > 0.05).

### Delay

The main effect of “target size” was not significant for the delay (*p* > 0.05). The main effect of group was found to be significant [*F*(2, 11) = 555.19, *p* < 0.0001, η*p*^2^ = 0.981; Figure 2C]. The delay was longer for the VPD when compared to HC participants (85 ± 12 vs. 73 ± 8 ms; *p* < 0.05). But it was shorter when compared to that exhibited by the IPD group (246 ± 23 ms; *p* < 0.05). When comparing the IPD and the HC groups a significant difference did emerge (246 ± 23 vs. 73 ± 8 ms; *p* < 0.05). No significant two-way interaction “target size” by “group” was found (*p* > 0.05).

## Discussion

The aim of this study was to compare the kinematic patterning of patients diagnosed with VPD and IPD during a reach-to-grasp task. The results indicate that patients with VPD showed similar movement durations and hand-grip conformation to patients with IPD, but longer movement duration and smaller hand opening than controls. Furthermore, for patients with VPD the onset of the grasping component was delayed with respect to the onset of the transport component when compared to the performance of controls. Although this pattern has been retrieved also for the patients with IPD, the VPD group showed a significantly shorter delay. Interestingly, the standard prehension task provides a simple and natural opportunity to examine whether the organization of upper-limb movements is somewhat dysfunctional in patients with VPD. The nature of this task, composed of a proximal transport component and a distinct but inter-related distal manipulation constituent, makes it a potentially good candidate for the exploration of the motor consequences of the disorder. This view is also supported by empirical evidence suggesting that subthalamic nucleus and internal pallidum overactivity is responsible for motor-related deficit in VPD (42) and that in primates the pallidal output of the basal ganglia is directed toward the ventrolateral thalamus, which selectively innervates the hand representation in the primary motor cortex (43, 44). Nevertheless this assumption should be taken with a certain degree of caution, given that the putative pathophysiology of VPD varies according to the type of evidence found and the behavioral manifestations observed can be linked to lesions at any level of the cortico-subcortical motor loops (45).

Zooming on the results of the kinematic analysis, significantly different patterns were found for the two target sizes in all the groups studied. The movement time was longer and the maximum grip aperture was reduced for smaller as compared to larger targets in both groups of patients (19, 24) as well as in the neurologically healthy participants (8–10). Thus, patients with VPD, as for the other groups, were able to modify the spatiotemporal characteristics of the grasping pattern in response to experimentally imposed changes in the size of the object. Patients with VPD showed longer movement duration for actions requiring greater accuracy such as when reaching for smaller objects (8–10). And they were able to scale hand opening in relation to the size of the object to be grasped (8–10). It appears, therefore, that VPD does not necessarily lead to any significant impairment of the central processes involved in organizing the reach-to-grasp movement.

Patients with VPD took longer to complete the movement and reached a smaller peak aperture than age-matched control participants. Similarly, and as previously demonstrated, patients with IPD demonstrated that their reach-to-grasp movements were slower (19, 24) and their maximum grip aperture smaller (41) with respect to control participants. Thus, VPD patients do show bradykinesia and hand hypometria, which limits the speed of movement execution and affects the modulation of hand aperture, respectively. This suggests that, as reported for patients with IPD, patients with VPD might have problems modulating movement speed and the command related to the opening/closing phases of the hand.

The kinematic analysis of the reach-to-grasp task allows examining the hypothesis that Parkinson’s disease leads to a problem with concurrent execution of functionally independent motor programs with the same limb (19, 46). In this respect, significant grasp-transport coordination impairments have been observed (19, 47). On average, IPD patients tended to start distancing the index finger and the thumb later than control subjects, relative to the onset of the transport movement (i.e., delay). It appears, therefore, that IPD does lead to a significant impairment of the central processes involved in organizing the concurrent execution of functionally independent motor programs, which are executed by the same effector system. It is possible that the disease affects the well-established motor programs controlling the coordination of subcomponents in the performance of everyday actions such as reaching and grasping.

Here, we found that also patients with VPD started to open the hand later than controls. A point worth noting, however, is that the extent of the delay between the transport and the manipulation components was less for the patients with VPD than for patients with IPD, resembling the delay exhibited by the control participants. Nevertheless, this effect but might be the result of the same mechanism, namely the difficult coordination of movements with a motor output system – disrupted by pathological descending signals – which significantly limit the ability to assemble movement components. Tentatively, we suggest that lesions linked to VPD motor outcomes may affect the responsiveness of cortical areas to activation – defined as the readiness to the elaboration of triggers not originating from the basal ganglia – and result in an inadequate cortical preparation of the movement. If this lack of cortical responsiveness was confined to a specific neural channel (e.g., reach or grasping), this would explain why a movement shows a delay of activation. The different pattern of results might indicate that the more focal pathophysiology resulting in VPD less affects this cortical readiness phenomenon. The ultimate reason why this is so, still remains to be determined.

We are fully aware that the present study has some limitations. Indeed, VPD encompasses a heterogeneous set of conditions and the extent of the spectrum of VPD remains quite imprecise. However, given the promising results in finding markers differentiating VPD and IPD kinematical profiles, further work should address a full characterization of the unfolding of the reach-to-grasp movement in this population.

In conclusion, the present study provides the first attempt to compare the kinematic patterning of reach-to-grasp movements in VPD with respect to the better characterized IPD, in the effort of unveiling possible upper-limb dysfunctions in this population. The results indicate that the basic pattern of performance is similar across the two groups of patients. They both show bradykinesia, hypometria, and loss of coordination between the reach and the grasp components. However, the dysfunction in the concurrent execution of the coordinated motor plans, patients with VPD appear to be much less compromised than patients with IPD. With a certain degree of caution, we contend that this kinematical landmark might be a useful tool for distinguishing across different parkinsonian syndromes.

## Conflict of Interest Statement

The authors declare that the research was conducted in the absence of any commercial or financial relationships that could be construed as a potential conflict of interest.
